# Prevalence and determinants of oral infection by Human Papillomavirus in HIV-infected and uninfected men who have sex with men

**DOI:** 10.1371/journal.pone.0184623

**Published:** 2017-09-14

**Authors:** Francesca Rollo, Alessandra Latini, Barbara Pichi, Manuela Colafigli, Maria Benevolo, Ilenia Sinopoli, Isabella Sperduti, Valentina Laquintana, Giulia Fabbri, Mirko Frasca, Antonio Cristaudo, Massimo Giuliani, Maria Gabriella Donà

**Affiliations:** 1 Pathology Department, Regina Elena National Cancer Institute, IFO, IRCCS, Rome, Italy; 2 STI/HIV Unit, San Gallicano Dermatologic Institute, IFO, IRCCS, Rome, Italy; 3 Otolaryngology Head Neck Surgery Department, Regina Elena National Cancer Institute, IFO, IRCCS, Rome, Italy; 4 Biostatistics Unit, Regina Elena National Cancer Institute, IFO, IRCCS, Rome, Italy; Fondazione IRCCS Istituto Nazionale dei Tumori, ITALY

## Abstract

**Background:**

Oral Human Papillomavirus (HPV) infection is rare in the general population but common in high-risk individuals. Recent data indicate that oral HPV is associated with the development of head and neck carcinomas. HPV16 infection, in particular, increases the risk of oropharyngeal cancer.

**Methods:**

We evaluated oral HPV prevalence and determinants of infection in cancer-free HIV-infected and uninfected men who have sex with men (MSM) recruited among attendees of an STI/HIV centre. Oral rinse and gargles were collected using a mouthwash and analyzed with the Linear Array HPV Genotyping Test. Socio-demographic and behavioral data were collected through face-to-face interviews.

**Results:**

Overall, 170 MSM participated: 98 HIV-uninfected and 72 HIV-infected (91.7% under cART). Oral HPV was detected in 17.3% and 27.8% of the subjects, respectively (p = 0.13). Non-carcinogenic HPVs were significantly more common among HIV-infected MSM (18.1% vs. 5.1%, p = 0.01). Prevalence of the HPV types included in the quadrivalent HPV vaccine was similar (6.1% vs. 8.3% for the HIV-negative and positive MSM, respectively, p = 0.76). HPV16 was the most frequent type in HIV-negative (5.1%), and HIV-positive individuals, in the latter group together with HPV18, 72 and 84 (4.2% each). Older age at first sex (AOR: 4.02, 95% CI: 1.17–13.86 for those older than 18 years of age at first intercourse, p = 0.027) and a higher lifetime number of receptive oral sex partners (AOR: 9.14, 95% CI: 2.49–33.62 for those with >50 compared to ≤50 partners, p<0.001) were determinants of oral HPV among HIV-infected MSM.

**Conclusion:**

Oral HPV infection among MSM attending an urban STI center is very frequent compared to the general population. Sexual behavior appears to be the major determinant of infection among the HIV-infected individuals.

## Introduction

Oral infection by Human Papillomavirus (HPV) increases risk for the development of oropharyngeal squamous cell carcinoma (OPSCC). HPV16 represents the genotype involved in the etiology of the majority of HPV-related OPSCC. Remarkably, oral HPV16 infection is associated with a 22-fold increased risk of incident OPSCC [1].

Because of the role of HPV in the development of orophrayngeal cancer and the steady increase in the incidence of this neoplasia observed in several countries [2–4], interest in the epidemiology of oral HPV infection has been growing. This is an uncommon infection in the general population, as shown by large studies conducted on healthy individuals in the US [5] and China [6]. Gillison and collaborators found a prevalence of 6.9% for any HPV and 1% for HPV16^5^. They also showed that oral infection is more frequent in men than women (10.1% vs. 3.6%). Being male is in fact an established risk factor for prevalent oral HPV [5,7].

Sexual behavior plays a major role in the acquisition of oral HPV infections and changes in sexual activity are probably contributing to the increase in the incidence of HPV-positive OPSCC, together with changes in smoking habits, particularly among some populations. Importantly, specific sexual practices (i.e., receptive oral sex) that may enhance the exposure of oropharyngeal mucosa to HPV infection represent important risk factors for HNSCC [8,9]. A greater number of partners for any and oral sex, a younger age at sexual debut and ever having had oral sex have been shown to be associated with risk of orophrayngeal cancer, despite the fact that differences have been found related to cancer subsite and gender [9]. Notably, same sex sexual contact has been shown to be associated with an almost 9-fold increased risk for base of the tongue cancer in men [9]. Due to their sexual practices, men who have sex with men (MSM), who also harbor the highest prevalence of anal HPV [10,11], show a significantly higher prevalence of oral HPV infection than the general population. Although data are very limited, their risk of HNSCC also seems to be higher in these subjects, as shown by a Danish study [12]. Notably, HIV-positive individuals harbor up to a 6-fold increase in risk for both oral HPV infection and HNSCC compared to their HIV-uninfected counterparts [13–15]. Although the data available on the natural history of oral HPV infection are very scarce, a higher incidence, however, has been reported for HIV-positive compared to HIV-negative MSM [16,17].

Despite the fact that the epidemiology of oral HPV in high-risk populations has been increasingly investigated, only few studies have been conducted in Europe [18–20]. Sparse data obtained have been gained in Italy but only on small to moderately-sized groups of MSM [21–23]. It is thus important to gather new data on this high-risk population.

We conducted a cross-sectional study to evaluate oral HPV infection in HIV-infected and uninfected cancer-free MSM attending an STI/HIV centre, and to investigate the determinants of infection in the two groups.

## Materials and methods

### Study population

From November 2014 to May 2016, all MSM attendees of the STI/HIV centre of the San Gallicano Dermatological Institute (Rome, Italy) were asked to participate in the OHMAR (Oral/Oropharyngeal HPV in Men At Risk) study. Individuals that met the following inclusion criteria were included: 1. age ≥18 years; 2. sexual intercourse with a man in the preceding 6 months; 3. no history of HNSCC; 4. no clinically suspicious lesions for HNSCC, as ascertained during a full otolaryngology examination conducted at enrollment. HIV-infected MSM were also included in the study. Face-to-face interviews were used to collect socio-demographic data and risk factor information. Clinical, virological and immunological data of the HIV-infected patients were retrieved from their medical records. A written informed consent was obtained from the participants. All procedures were performed in accordance with the Helsinki Declaration. The study was cleared by the institutional Ethics Committee, I.F.O. Section (Istituti Regina Elena e San Gallicano)–Fondazione Bietti (CE/417/14).

### Oral sample collection

Oral specimens were collected using 15 ml of Listerine mouthwash. Participants were asked to alternatively rinse and gargle for a total time of 30 seconds. Samples were immediately placed on ice, and within 15 min, centrifuged for 10 min at 3000xg, 4°C. The supernatant was removed and the pellet was washed twice with PreservCyt (Hologic, Pomezia, Italy). Subsequently, the pellet was resuspended in 2 ml of PreservCyt.

### HPV testing

The Linear Array HPV genotyping test (Roche Diagnostics, Milan, Italy), which detects 37 genotypes (HPV 6, 11, 16, 18, 26, 31, 33, 35, 39, 40, 42, 45, 51, 52, 53, 54, 55, 56, 58, 59, 61, 62, 64, 66, 67, 68, 69, 70, 71, 72, 73, 81, 82, 83, 84, CP6108 and IS39) was used. Total nucleic acids were extracted from 250 μl of the PreservCyt sample using the Amplilute Liquid Media Extraction kit, part of the Linear Array HPV genotyping test, following the manufacturer’s instructions. 50 μl of the eluate was used for amplification. Automated detection was performed in a Profiblot T48 (Tecan, Männedorf, Switzerland). Results were interpreted as indicated by the producer. Samples with presence of one or more HPV hybridization bands, independently from the presence of the internal control, represented by ß-globin, were considered as positive.

### Data analysis

Descriptive statistics were computed for all the variables of interest in order to provide summarized descriptions of the study population. Regarding drinking habits, participants were asked to indicate their consumption of wine, beer, and spirits separately: 350 ml of beer, 120 ml of wine, and 50 of spirits were considered as one dose of alcoholic beverage (10–12 g of alcohol). Study subjects were then classified as follows: non-drinker (no alcohol consumption); light drinker (≤1 dose per day); moderate drinker (up to 3 doses per day); heavy drinker (>3 doses per day). Frequency of receptive oral sex was classified as follows: occasionally (<1 time per week), often (1–2 times per week), very often (≥3 times per week). The Mann-Whitney test was used for comparisons between median values. Socio-behavioral characteristics and lifestyle habits of HIV-positive and HIV-negative individuals were compared using Chi-square tests.

Participants were considered positive for any HPV whenever at least one of the 37 types detectable by the Linear Array was evidenced in the oral rinse. HPV genotypes were considered as carcinogenic, possibly carcinogenic, or non-carcinogenic based on the risk assigned to each genotype by the International Agency for Research on Cancer [24], and as previously specified [25], with the following modifications for purposes of analysis: HPV68, classified by IARC as a probably carcinogenic type, was included in the carcinogenic HPV group; HPV 55, 62, 64, 71, 83 and 84, classified by IARC as types with undetermined risk, were considered as non-carcinogenic types. The final HPV risk classification used in this study was: i) carcinogenic: HPVs 16, 18, 31, 33, 35, 39, 45, 51, 52, 56, 58, 59, 68; ii) possibly carcinogenic: HPVs 26, 53, 66, 67, 69, 70, 73, 82; iii) non-carcinogenic: HPVs 6, 11, 40, 42, 54, 55, 61, 62, 64, 71, 72, 81, 83, 84, CP6108, IS39. Participants were considered positive for the types of the quadrivalent HPV vaccine (qHPV), if any of the 4 types included in the vaccine, i.e., HPVs 6, 11, 16, 18, was detected in the oral sample.

To assess the relationship between categorical variables, we used the Pearson’s Chi-square test and the Fisher Exact test, depending on the size of groups compared. A multivariate logistic regression model was generated for each of the two study groups. Both for the HIV-negative and positive MSM, the final model included: i) all the variables significant in the univariate assessment for any of the groups (p value <0.05): age at first sex with a man, number of lifetime partners for any sex and receptive oral sex, number of recent partners for any sex and receptive oral sex, history of ano-genital warts; ii) covariates known to be associated with oral HPV based on the literature, regardless of their significance in the univariate analysis: age, smoking status, occasional partners for any sex and receptive oral sex. The multivariate logistic regression models were built using a stepwise regression approach (forward selection) and the related estimates reported as Adjusted Odds Ratio (AOR) and 95% Confidence Interval (CI). The enter and remove limits were p = 0.10 and p = 0.15, respectively. Statistical analyses were carried out using SPSS software (SPSS version 21, SPSS Inc., Chicago, IL, USA).

## Results

### Study population

During the study period, a total of 170 MSM, mostly Caucasian (164, 96.5%), were recruited for this cross-sectional study: 98 (57.6%) HIV-uninfected and 72 HIV-infected individuals (42.4%).

The main socio-demographic and behavioral characteristics of the participants are shown in Table 1. The median age was 39 years (IQR: 32–45) for the HIV-negative and 44 years (IQR: 39–49) for the HIV-positive MSM (p = 0.04). Both groups of individuals had a similar median age at sexual debut (p = 0.13). Median numbers of lifetime partners for any sex (p = 0.87) and receptive oral sex (p = 0.56) were similar for both groups. Differently, HIV-infected MSM had a significantly lower number of recent partners both for any sex (p<0.001) and receptive oral sex (p = 0.005). HIV-negative and HIV-positive MSM were significantly different regarding education, alcohol consumption, partnership for any sex and receptive oral sex, and STI history. All the HIV-negative participants and 98.6% of the HIV-infected MSM reported to have engaged in receptive oral sex in their life, and the majority declared to have practiced receptive oral sex in the preceding 6 months (93.9% and 88.9%, respectively). Almost all the participants declared that they practiced condomless receptive oral sex both with their steady partner and with their occasional partners.

**Table 1:** Socio-demographic, behavioral and lifestyle characteristics at enrollment of the study population of 170 HIV-uninfected and infected MSM.

	HIV-uninfected MSM		HIV-infected MSM		
	N = 98		N = 72		
	median (IQR)				*p* value
**Age, years**	39 (32–45)		44 (33–49)		**0.04**
**Age at first sex with a man, years**	19 (17–24)		18 (17–20)		0.13
**N. lifetime partners for any sex**	87 (45–150)		100 (32–300)		0.87
**N. recent partners for any sex**	8 (4–16)		2 (1–9)		**<0.001**
**N. lifetime partners for receptive oral sex**	50 (20–95)		50 (15–150)		0.56
**N. recent partners for receptive oral sex**	4 (2–10)		2 (1–6)		**0.005**
	**n**	**%**	**n**	**%**	***p* value**
**Graduate education**	51	52.0	22	30.6	**0.005**
**Medium/high income**^**a**^	63	64.3	45	62.5	0.81
**Alcohol consumption**					**0.02**
**non drinker**	40	40.8	40	55.6	
**light drinker**	20	20.4	16	22.2	
**moderate drinker**	34	34.7	14	19.4	
**heavy drinker**	4	4.1	2	2.8	
**Smoking status**					0.06
**never**	58	59.2	31	43.1	
**former**	5	5.1	7	9.7	
**current**	35	35.7	34	47.2	
**Partnership for any sex**					**0.0004**
**only steady partner**	3	3.1	13	18.1	
**steady and occasional partners**	24	24.5	22	30.6	
**only occasional partners**	71	72.4	37	51.4	
**Receptive oral sex (ever)**	98	100.0	71	98.6	0.24
**Recent receptive oral sex**	92	93.9	64	88.9	0.24
**Frequency of recent receptive oral sex**					0.29
**occasionally**	60	65.2	39	60.9	
**often**	30	32.6	20	31.3	
**very often**	2	2.2	5	7.8	
**Partnership in recent receptive oral sex**					**<0.0001**
**only steady partner**	5	5.1	19	26.4	
**steady and occasional partners**	15	15.3	13	18.1	
**only occasional partners**	72	73.5	32	44.4	
**Condomless receptive oral sex with steady partner**^**b**^	19	95.0	32	100.0	0.21
**Condomless receptive oral sex with occasional partners**^**c**^	72	82.8	34	75.6	0.32
**STI history**^**d**^	67	68.4	59	81.9	**0.047**
**Ano-genital wart history**^**e**^	30	30.6	28	38.9	0.26

STI, Sexually Transmitted Infections.

Significant differences are highlighted in bold.

^a^ Income > 12,000 €/year.

^b^ For the participants with a steady partner (n = 20 for HIV-uninfected MSM; n = 32 for HIV-infected MSM).

^c^ For the participants with occasional partners (n = 87 for HIV-uninfected MSM; n = 45 for HIV-infected MSM).

^d^ Ano-genital warts, genital herpes, syphilis, gonorrhea (any site), diagnosed at least 6 months prior to enrollment.

The 72 HIV-infected participants had a median nadir CD4+ count of 300 cells/mm3 (IQR: 200–391). At enrollment, the median CD4+ count was 622 cells/mm3 (IQR: 479–817). Only 4 patients (5.5%) had received a diagnosis of AIDS. The majority of the HIV-positive individuals were on cART (66, 91.7%); almost all the cART-experienced MSM (60, 90.9%) were aviremic at enrollment.

### Overall and type-specific oral HPV prevalence

A valid HPV test result was obtained for all the samples analyzed. Overall, HPV was detected in 37 of the 170 collected oral rinses (21.7%). Specifically, HPV infection was found in 17.3% and 27.8% of the HIV-negative and positive MSM, respectively (p = 0.13) (Table 2). No significant difference in the proportion of individuals positive for oncogenic types was observed for the two study groups, while non-oncogenic HPVs were significantly more common among HIV-infected subjects (18.1% vs. 5.1%, p = 0.01). Similar proportions of individuals were positive for the HPV types included in the quadrivalent HPV vaccine (p = 0.76). Multiple infections were twice as common among HIV-infected MSM, although this difference did not reach statistical significance (9.7% vs. 4.1%, p = 0.21). In HIV-positive individuals, up to 6 different types were found, compared to 4 types maximum for their HIV-negative counterparts (data not shown).

**Table 2:** Distribution of oral HPV infection in the 170 study participants, stratified according to HPV group and HIV status.

	HIV-uninfected MSM N = 98		HIV-infected MSM N = 72		*p* value
	n	%	n	%	
**Any HPV**	17	17.3	20	27.8	0.13
**Carcinogenic HPV**^**a**^	9	9.2	8	11.1	0.79
**Possibly carcinogenic HPV**^**b**^	7	7.1	7	9.7	0.58
**Non-carcinogenic HPV**^**c**^	5	5.1	13	18.1	**0.01**
**qHPV**^**d**^	6	6.1	6	8.3	0.76
**>1 HPV genotype**	4	4.1	7	9.7	0.21

Significant differences are highlighted in bold.

^a^ HPVs 16, 18, 31, 33, 35, 39, 45, 51, 52, 56, 58, 59, 68.

^b^ HPVs 26, 53, 66, 67, 69, 70, 73, 82.

^c^ HPVs 6, 11, 40, 42, 54, 55, 61, 62, 64, 71, 72, 81, 83, 84, CP6108, IS39.

^d^ HPVs 6, 11, 16, 18.

**Fig 1:**
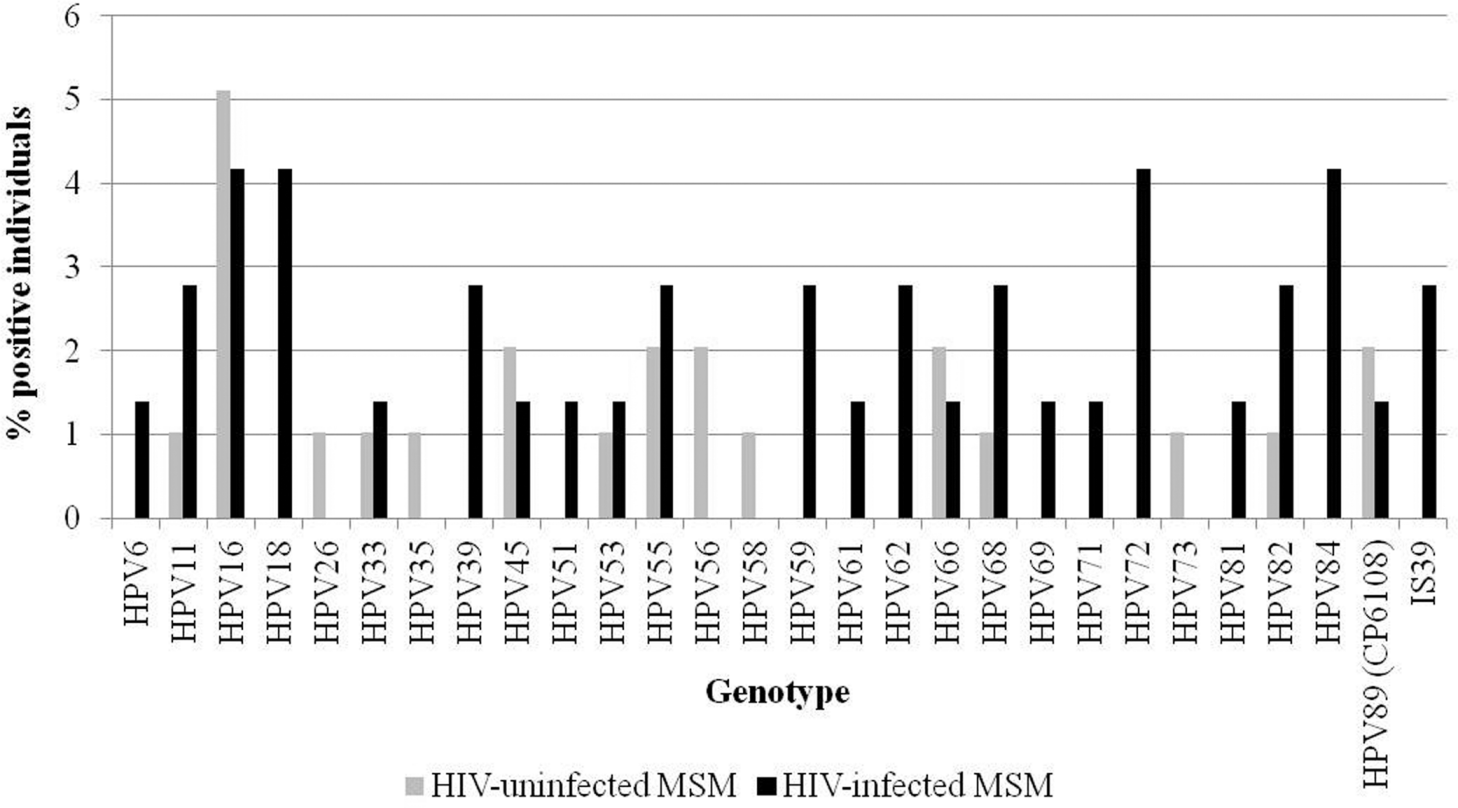
Distribution of the HPV genotypes detected in the 170 oral samples collected from MSM. Prevalence of each genotype is shown for the 72 HIV-infected and 98 HIV-uninfected participants.

A total of 23 different HPV types were detected among HIV-infected MSM, while only 15 genotypes were found in the HIV-uninfected participants. Type-specific prevalence of all the genotypes found is shown in Fig 1. HPV16 was the most frequent type in HIV-negative MSM (5.1%). Among the HIV-positive individuals, HPV16, 18, 72 and 84 all showed the highest prevalence (4.2% each).

### Determinants of oral HPV infection

Associations between oral HPV infection by any HPV genotype and sexual behavior, STI history, oral health, and lifestyle (smoking and drinking habits) were investigated separately for each study group. The results of the univariate and multivariate analyses by HIV status are shown in Table 3. Subset analyses for carcinogenic HPVs and HPV16 were not performed due to the limited number of positive individuals observed in each study group.

**Table 3:** Univariate and multivariate analyses of the association between oral HPV infection (any HPV), socio-demographic factors, sexual behavior and HIV parameters.

	HIV-uninfected MSM					HIV-infected MSM				
	HPV-positive	COR (95% CI)	*p* value	AOR (95% CI)	*p* value	HPV-positive	COR (95% CI)	*p* value	AOR (95% CI)	*p* value
	n/N (%)					n/N (%)				
**Age, years**										
<40	9/54 (16.7)	Ref				5/30 (16.7)	Ref			
≥40	8/44 (18.2)	1.11 (0.39–3.17)	0.84	not retained^a^		15/42 (35.7)	2.78(0.88–8.76)	0.08	not retained^a^	
**Education**										
graduate	9/51 (17.6)	Ref				6/22 (27.3)	Ref			
ungraduate	8/47 (17.0)	0.96 (0.34–2.73)	0.94			14/50 (28.0)	1.04 (0.34–3.19)	0.95		
**Annual income**										
medium/high	12/63 (19.0)	Ref				13/45 (28.9)	Ref			
low	5/35 (14.3)	0.71 (0.23–2.21)	0.55			7/27 (25.9)	0.86 (0.29–2.52)	0.79		
**Alcohol consumption**										
no/light	9/60 (15.0)	Ref				17/56 (30.4)	Ref			
moderate/heavy	8/38 (21.1)	1.51 (0.53–4.33)	0.44			3/16 (18.8)	0.53 (0.13–2.10)	0.37		
**Smoking status**										
never/former	10/63 (15.9)	Ref				9/38 (23.7)	Ref			
current	7/35 (20.0)	1.32 (0.45–3.86)	0.61	not retained^a^		11/34 (32.4)	1.54 (0.55–4.35)	0.41	not retained^a^	
**Age at first sex with a man, years**										
≤18	9/43 (20.9)	Ref				6/37 (16.2)	Ref			
>18	8/55 (14.5)	0.64 (0.22–1.84)	0.41	not retained^a^		14/35 (40.0)	**3.44 (1.14–10.40)**	**0.028**	**4.02 (1.17–13.86)**	**0.027**
**N. lifetime partner for any sex**										
<100	12/54 (22.2)	Ref				5/34 (14.7)	Ref			
≥100	5/44 (11.4)	0.45 (0.14–1.39)	0.16	not retained^a^		15/38 (39.5)	**3.78 (1.20–11.95)**	**0.019**	not retained^a^	
**N. recent partner for any sex**										
≤4	7/31 (22.6)	Ref				8/43 (18.6)	Ref			
>4	10/67 (14.9)	0.60 (0.20–1.77)	0.35	not retained^a^		12/29 (41.4)	**3.09 (1.06–8.97)**	**0.03**	not retained^a^	
**N. lifetime partners for receptive oral sex**										
≤50	12/57 (21.0)	Ref				4/39 (10.2)	Ref			
>50	5/41 (12.2)	0.52 (0.17–1.61)	0.25	not retained^a^		16/33 (48.5)	**8.23 (2.38–28.44)**	**<0.001**	**9.14 (2.49–33.62)**	**<0.001**
**N. recent partners for receptive oral sex**										
≤4	12/52 (23.1)	Ref				9/47 (19.1)	Ref			
>4	5/46 (10.9)	0.40 (0.13–1.26)	0.11	not retained^a^		11/25 (44.0)	**3.32 (1.13–9.70)**	**0.02**	not retained^a^	
**Occasional partner for any sex**										
no	1/3 (33.3)	Ref				2/19 (10.5)	Ref			
yes	16/95 (16.8)	0.40 (0.03–4.74)	0.47	not retained^a^		18/53 (34.0)	4.37 (0.91–21.04)	0.07	not retained^a^	
**Occasional partner for receptive oral sex**^**c**^										
no	0/5 (0.0)	Ref				3/19 (15.8)	Ref			
yes	13/87 (14.9)	1.99 (0.10–38.18)	0.64	not retained^a^		19/64 (29.7)	2.94 (0.74–11.64)	0.12	not retained^a^	
**Frequency recent receptive oral sex**^**c**^										
occasionally	11/60 (18.3)	Ref				9/39 (23.1)	Ref			
often/very often	2/32 (6.3)	0.29 (0.06–1.43)	0.13			10/25 (40.0)	2.22 (0.74–6.63)	0.15		
**Condomless receptive oral sex**										
no	0/2 (0.0)	Ref				1/4 (25.0)	Ref			
yes	13/90 (14.4)	1.15 (0.05–25.26)	0.93			18/60 (30.0)	1.29 (0.12–13.21)	0.83		
**STI history**^**d**^										
no	5/31 (16.1)	Ref				3/13 (23.1)	Ref			
yes	12/67 (17.9)	1.13 (0.36–3.56)	0.83			17/59 (28.8)	1.35 (0.33–5.51)	0.68		
**Ano-genital warts history**										
no	8/68 (11.8)	Ref				14/44 (31.8)	Ref			
yes	9/30 (30.0)	**3.21 (1.10–9.41)**	**0.033**	not retained^a^		6/28 (21.4)	0.58 (0.19–1.76)	0.34	not retained^a^	
**Presence of tonsils**										
no	5/25 (20.0)	Ref				3/12 (25.0)	Ref			
yes	12/73 (16.4)	0.79 (0.25–2.51)	0.68			17/60 (28.3)	1.19 (0.29–4.92)	0.81		
**Gum blooding (ever)**										
no	8/60 (13.3)	Ref				17/56 (30.4)	Ref			
yes	9/38 (23.7)	2.02 (0.70–5.79)	0.19			3/16 (18.8)	0.90 (0.13–2.10)	0.53		
**Dental abscesses (ever)**										
no	15/78 (19.2)	Ref				13/55 (23.6)	Ref			
yes	2/20 (10.0)	0.47 (0.09–2.23)	0.34			7/17 (41.2)	2.26 (0.72–7.13)	0.16		
**Toothache (ever)**										
no	13/69 (18.8)	Ref				12/47 (25.5)	Ref			
yes	4/29 (13.8)	0.69 (0.20–2.32)	0.55			8/25 (32.0)	1.37 (0.47–3.98)	0.56		
**Tooth loss (ever)**										
no	12/74 (16.2)	Ref				15/55 (27.3)	Ref			
yes	5/24 (20.8)	1.36 (0.42–4.35)	0.60			5/17 (29.4)	1.11 (0.33–3.69)	0.86		
**Dental cleaning per year**										
≥1	16/83 (19.3)	Ref				13/56 (23.2)	Ref			
<1	1/15 (6.7)	0.30 (0.04–2.44)	0.26			7/16 (43.8)	2.57 (0.80–8.26)	0.12		
**Mounth wash use**										
often/always	7/44 (15.9)	Ref				6/26 (23.1)	Ref			
never/sometime	10/54 (18.5)	1.20 (0.42–3.47)	0.73			14/46 (30.4)	1.46 (0.48–4.41)	0.50		
**Oral hygiene**^**e**^										
good/very good	4/30 (13.3)	Ref				4/17 (23.5)	Ref			
very poor/poor/fair	6/34 (17.6)	1.39 (0.35–5.50)	0.64			12/32 (37.5)	1.95 (0.52–7.37)	0.32		
			**HIV-related parameters**			**HPV-positive**	**COR (95% CI)**	***p* value**	**AOR (95% CI)**	***p* value**
						**n/N (%)**				
			**Nadir CD4+ count (cells/mm3)**							
			≥200			14/54 (25.9)	Ref			
			<200			6/18 (33.3)	1.43 (0.45–4.53)	0.54		
			**Current CD4+ count (cells/mm3)**							
			≥500			13/52 (25.0)	Ref			
			<500			7/20 (35.0)	1.61 (0.53–4.91)	0.40		
			**HIV-RNA load (copies/ml)**							
			<40			18/61 (29.5)	Ref			
			≥40			2/11 (18.2)	0.53 (0.10–2.70)	0.44		
			**CDC stage**							
			A/B			17/68 (25.0)	Ref			
			C			3/4 (75.0)	9.00 (0.88–92.40)	0.06		
			**cART**							
			yes			20/67 (29.9)	Ref			
			no			0/5 (0.0)	0.21 (0.01–3.99)	0.30		

COR, Crude Odds Ratio; CI, Confidence Interval; AOR, Adjusted Odds Ratio; STI, Sexually Transmitted Infection; cART, combined anti-retroviral therapy.

Covariates used for the logistic regression: age, smoking status, age at first sex with a man, number of lifetime partners for any sex and receptive oral sex, number of recent partners for any sex and receptive oral sex, occasional partners for any sex and receptive oral sex, history of ano-genital warts

Significant associations are highlighted in bold.

^a^ Not retained in the final adjusted model

^b^ Low: <12,000 €; medium: 12,000–24,000 €; high: >24,000 €

^c^ Only the participants reporting receptive oral sex were included

^d^ Genital herpes, syphilis, gonorrhea (any site)

^e^ Available for 113 participants

HIV-uninfected MSM who reported a history of ano-genital warts were significantly more likely to harbor oral HPV infection (COR: 3.21, 95% CI: 1.10–9.41, p = 0.033).

Among the HIV-infected MSM, the odds of oral infection increased with age at first sexual intercourse (COR: 3.44, 95% CI: 1.14–10.40 for those older than 18 years at first sex, p = 0.028), the number of lifetime partners for any sex (COR: 3.78, 95% CI: 1.20–11.95, for those with ≥100 compared to <100 partners, p = 0.019) and for receptive oral sex (COR: 8.23, 95% CI: 2.38–28.44, for those with >50 compared to ≤50 partners, p<0.001). A significant increase in oral HPV prevalence was also observed among those who reported more than 4 recent partners for any sex (COR: 3.09, 95% CI: 1.06–8.97, p = 0.03) and receptive oral sex (COR: 3.32, 95% CI: 1.13–9.70, p = 0.02). HIV-positive MSM who referred to having sex with occasional partners also showed a higher prevalence of infection (COR: 4.37, 95% CI: 0.91–21.04, p = 0.066). Oral HPV was more frequent among HIV-infected MSM of more than 40 years of age (COR: 2.78, 95% CI: 0.88–8.76, p = 0.08). Regarding the HIV parameters, a borderline association was observed with the CDC-93 stage. In fact, oral HPV infection was more frequent among individuals in stage C compared to those in stage A/B (COR: 9.00, 95% CI: 0.88–92.40, p = 0.06).

Neither factors associated with oral health nor presence of tonsils appeared to modify the risk for oral HPV infection in the two study groups. After the beginning of the study, the otolaryngologist who performed the clinical examination was asked to rate the oral hygiene of the participants. This evaluation was available for 113 individuals. A non-significant increase in oral HPV infection was found for MSM with very poor/poor/fair hygiene.

For the HIV-uninfected MSM, none of the covariates was retained in the final adjusted model. For the HIV-infected MSM, an independent association for oral infection by any HPV was observed with age at first sex with a man (AOR: 4.02, 95% CI: 1.17–13.86, p = 0.027 for those reporting to be older than 18 years at first intercourse) and number of lifetime receptive oral sex partners (AOR: 9.14, 95% CI: 2.49–33.62, p<0.001 for those with >50 partners).

## Discussion

This cross-sectional study provided data on the overall and type-specific prevalence of oral HPV infection in HIV-infected and uninfected MSM. It also evidenced differences in the correlates of infection for these two groups of individuals. To the best of our knowledge, this is the largest Italian study on this topic. Epidemiological data on oral HPV infection may be useful to evaluate the potential impact of HPV vaccination on MSM, although very few data on the efficacy of the HPV vaccines against oral infection have been provided to date. The Costa Rica Trial has shown a reduced prevalence in the vaccinated women compared to the control group [26], but we still lack data on the vaccine efficacy in HNSCC prevention.

In this study, we used oral rinse and gargles for the assessment of HPV infection. This type of sample, notwithstanding the inability to reveal the specific site of infection, has been shown to be better than other specimens for oral HPV detection [20,21,27,28].

Our estimates for the prevalence of any HPV among HIV-negative and HIV-positive MSM are 4 to 7-fold higher in comparison with the prevalence observed in young adults recruited in Italy [29], while they are similar to those found in other comparable cohorts of MSM [21,27]. Moreover, they almost perfectly overlap the pooled prevalence estimated for these individuals by a recent meta-analysis, i.e., 17.1% and 28.9%, respectively [30]. Similarly, the prevalence of carcinogenic HPV infection in HIV-negative MSM (9.2%) is very close to the pooled prevalence of this meta-analysis (9.1%), while the corresponding figure for HIV-positive individuals (11.1%) is lower than that estimated by King and collaborators (16.5%).

HPV16 was the most prevalent genotype in both study groups, confirming that HPV16 is the most common genotype in oral infections, besides being by far the most frequent type in HPV-associated orophrayngeal cancers [31]. Nevertheless, many other different HPVs were found in our participants, particularly among HIV-infected subjects, who also more frequently harbored multiple infections.

Our study confirmed that oral HPV is more common among MSM with HIV infection. Although statistical significance was not reached, others have shown that HIV status is independently associated with oral HPV [18,27]. The difference observed in our study does not seem to be attributable to a greater exposure to HPV for HIV-infected subjects through high risk sexual habits, since they were less likely to report receptive oral sex with occasional partners and had a lower number of recent partners both for any sex and receptive oral sex compared to their HIV-negative counterparts. However, it is noteworthy that the HIV-negative were significantly younger than the HIV-positive participants and this may partly explain our findings. In fact, some studies showed that oral HPV prevalence increases with age among HIV-negative men [32], and among HIV-negative MSM [18]. Additionally, we observed a higher prevalence of infection among HIV-positive participants who were more than 40 years of age, while other studies did not evidence any age effect for these subjects [18,32]. We found that HIV-positive individuals were significantly more likely to have non-carcinogenic HPVs, consistently with other studies [18, 32].

We investigated socio-demographic variables, lifestyle, oral health and sexual habits in order to assess the determinants of oral HPV infection in the two study groups. We did not observe any association with recent oral sex behavior for the HIV-negative MSM, differently from other studies [32]. In the univariate analysis, a significant association was only found with having had ano-genital warts in the past. Consistently with previous studies [27,33], individuals reporting a history of ano-genital condylomatosis appeared to be at increased risk for prevalent oral HPV infection. In the multivariate analysis, a significant association was not evidenced for any of the factors investigated.

Number of lifetime partners for oral sex emerged as a determinant of prevalent oral HPV infection among HIV-infected MSM, in line with previous studies [7,27,32,34,35]. In particular, participants with more than 50 partners showed an almost 10-fold increased odds of oral HPV. Interestingly, we also observed a positive association with age at first intercourse. The odds for oral HPV increased 4-fold in those who reported an older age at first sexual experience with a man. Our observation may reflect a measurable age-cohort effect on age at first intercourse, due to the older age of the HPV-positive patients. Moreover, it must be noted that oral sex experiences may precede sexual debut.

This investigation failed to evidence a role for several other variables known to be associated with the risk for oral HPV infection, such as oral sex [7,34] and tobacco use, which has been demonstrated as a risk factor in men, MSM [18,27,32,35,36], as well as in the general population [5,7]. Regarding sex habits, the high frequency of risky behavior among MSM often limits the possibility of finding significant associations. For instance, we could not establish whether or not oral sex is associated with HPV infection, since very few individuals denied having ever practiced or having recently practiced receptive oral sex. Unfortunately, we did not collect data on oral-anal contacts, which may also play a role in the acquisition of oral HPV infection. Indeed, a previous study observed that oral HPV prevalence increased significantly with the number of rimming partners among HIV-negative MSM [32].

We also investigated the possible role of oral hygiene, since a large cross-sectional study has previously identified four different indicators of scarce oral health as being associated with increased likelihood of oral HPV [7], and poor oral hygiene also represents a risk factor for head and neck carcinomas [37–39]. However, we did not observe any significant increase in the odds of oral HPV for MSM with poor oral hygiene in any of the two study groups.

As regards the HIV parameters, we observed that HIV-positive MSM in C-CDC 93 stage, i.e., subjects diagnosed with an AIDS-associated disease, had a higher prevalence of infection, at borderline significance. This finding seems to suggest that a severe immunocompromised status favors oral HPV infection. However, we did not observe any significant association with nadir and current CD4 counts, consistently with other studies [10,18,27,36,40]. Nevertheless, others have demonstrated increased risk of oral infection for HIV-positive individuals with lower nadir and current CD4 counts, with a particular association with HPV16 infection [27,32,33]. Being on cART did not seem to significantly affect the prevalence of oral HPV. Data from the literature have displayed conflicting results. A recent investigation showed no effect of cART initiation on oral HPV infection [41]. No significant differences in oral HPV prevalence between cART-naïve and -experienced subjects have been also observed by others [32,36].

Some limitations of the present study can be evidenced. Firstly, the analyses may have been underpowered due to the small size of the study groups. Secondly, the participants homogeneously reported a high risk sexual behavior, so our results may not be generalizable to all urban MSM. Finally, the group of HIV-infected MSM was largely lacking in individuals with severe immunosuppression and this may have limited the validity of the association measures between oral HPV infection and the parameters of immunodeficiency.

In conclusion, this study showed that the prevalence of oral HPV in MSM is 3–5 fold higher in comparison with the general population and the infection is particularly frequent even among aviremic and immunologically balanced HIV-infected MSM. However, the sample size needs to be increased to have reliable estimates for the predictors of this infection. Since no established screening programs for OPSCC exist, further studies are needed to clarify whether or not individuals with oral HPV infection have to undergo specific management.
